# Preventive care for physical activity and fruit and vegetable consumption: a survey of family carer expectations of health service delivery for people with a mental health condition

**DOI:** 10.1186/s12913-020-5059-0

**Published:** 2020-03-12

**Authors:** Jacqueline M. Bailey, Tara L. Clinton-McHarg, Paula M. Wye, John H. Wiggers, Kate M. Bartlem, Jennifer A. Bowman

**Affiliations:** 1 School of Psychology, Faculty of Science and Information Technology, The University of Newcastle, University Drive, Callaghan, NSW 2308 Australia; 2 Hunter Medical Research Institute, Clinical Research Centre, Lot 1 Kookaburra Circuit, New Lambton Heights, NSW 2305 Australia; 3 Population Health, Hunter New England Local Health District, Booth Building, Wallsend Health Services, Longworth Avenue, Wallsend, Wallsend, NSW 2287 Australia; 4 School of Medicine and Public Health, Faculty of Health and Medicine, The University of Newcastle, University Drive, Callaghan, NSW 2308 Australia

**Keywords:** Chronic disease risk behaviours, Caregiver, Mental illness, Health services, Fruit and vegetable consumption, Physical activity

## Abstract

**Background:**

Chronic disease is a leading cause of death globally, where inadequate fruit and vegetable consumption and inadequate physical activity are consistently implicated as key contributing risk factors for such diseases. People with a mental health condition are reported to experience a higher prevalence of such risks and experience an increased morbidity and mortality from resultant chronic disease. Despite guidelines identifying a need for services accessed by people with a mental health condition to provide care to address such health risk behaviours, sub-optimal care is frequently reported suggesting a need for innovative strategies to increase the provision of physical health care. An exploratory study was conducted to examine: 1) family carers’ expectations of care provision regarding fruit and vegetable consumption and physical activity by health and community services for people with a mental health condition; 2) carer’s own health risk behaviour status and perceptions of the influence of the health risk behaviours on mental health; and 3) possible associations of socio-demographic, clinical and attitudinal factors with carer expectations of care provision for fruit and vegetable consumption and physical activity.

**Methods:**

Family carers (*n* = 144) of a person with a mental health condition completed a cross-sectional survey. Participants were members of a mental health carer support organisation operating in New South Wales, Australia.

**Results:**

A high proportion of participants considered care for fruit and vegetable consumption and physical activity respectively should be provided by: mental health hospitals (78.5, 82.7%); community mental health services (76.7, 85.9%); general practice (81.1, 79.2%); and non-government organisations (56.2, 65.4%). Most participants perceived adequate fruit and vegetable consumption (55.9%), and physical activity (71.3%) would have a very positive impact on mental health. Carers who perceived adequate fruit and vegetable consumption and physical activity would have a positive impact on mental health were more likely to expect care for such behaviours from some services.

**Conclusions:**

The majority of participants expected care for fruit and vegetable consumption and physical activity be provided by all services catering for people with a mental health condition, reinforcing the appropriateness for such services to provide physical health care for clients in a systematic manner.

## Background

Physical chronic diseases, such as cardiovascular diseases, cancer, and diabetes, are a leading cause of death globally and contributed to 71% of deaths in 2017 [1, 2]. Chronic conditions such as overweight and obesity (conservatively measured by Body Mass Index [BMI]) were also estimated to account for 4.7 million deaths globally in 2017 and 148 million Disability Adjusted Life Years (DALYs, sum of years lived with a disability and years of life lost) [3]. In 2014, 38 to 40% of adults were overweight and 11 to 15% obese; with the worldwide prevalence of obesity nearly doubling between 1980 and 2014, [1] and continuing to increase [4]. Such prevalence estimates continue to increase, with 2016 prevalence estimates of obesity ranging from 20 to 36% among adults in Australia, United Kingdom, United States, Canada, and many European nations [4].

Compared to these global figures, the reported figures in Australia are somewhat higher, with physical chronic diseases contributing to 87% of deaths in 2015 [5]. The prevalence of diabetes in Australia has tripled over the last 25 years with 6.1% (1.2 million people) of the adult population reported to have the condition in 2015 [5]. A recent study of more than 10 million adults from 239 prospective studies found that Australian and New Zealand adults with a BMI above the ‘normal weight’ range (BMI > 25 kg/m2) had an increased risk of death from all causes of 31%, for each 5 kg/m2 increase in BMI [6]. Further, compared to people with a ‘normal weight’ BMI, life expectancy was reduced by 2–4 years for people with class I obesity (BMI = 30–34.99), and by 8–10 years for people with class III obesity (BMI = > 39.99) [7].

Inadequate physical activity and inadequate fruit and vegetable consumption are two modifiable health risk behaviours that are consistently implicated as contributing to the incidence of physical chronic disease and overweight and obesity [3, 8, 9]. Inadequate physical activity is a risk factor for the development of chronic diseases such as ischemic heart disease, stroke, diabetes mellitus, and cancer, as well as the development of conditions that contribute to these chronic diseases such as overweight, obesity and hypertension [3, 10]. Similarly, inadequate fruit and/or vegetable consumption are risk factors for the development of obesity, and chronic diseases such as: ischemic heart disease; stroke; diabetes mellitus; stomach, oesophageal, colorectal, and lung cancer [3, 8, 9, 11–13]. Additionally, the combination of multiple health risk behaviours increases the risk of developing chronic disease and resultant mortality [14, 15]. National guidelines vary slightly between countries regarding what is considered to be an inadequate level of physical activity or inadequate fruit and vegetable consumption [16–22]. In Australia, current national guidelines state that the following behaviours may place an individual at risk of developing a chronic disease: 1) consuming less than five vegetable or two fruit servings per day [23]; 2) engaging in less than 150 to 300 min of moderate intensity physical activity (e.g. brisk walking, golf) or 75 to 150 min of vigorous intensity physical activity (e.g. jogging, aerobics, digging), or an equivalent combination each week; and participating in muscle strengthening activities on less than 2 days each week [24].

The prevalence of overweight and obesity, [25–27] inadequate physical activity [28, 29] and inadequate fruit and vegetable consumption [28, 29] is higher among people with a mental health condition than those without. Internationally and in Australia, people with a mental health condition experience increased rates of physical chronic disease, and resultant morbidity, mortality and reduced life expectancy compared to people without a mental health condition [30–32]. Research among this population group utilises various terminology such as ‘mental illness’ or ‘mental disorder’, the term ‘mental health condition’ will be used throughout this study to define mental health conditions commonly experienced by individuals accessing adult mental health services; that is, categories of mental illness outlined in the Diagnostic and Statistical Manual of Mental Disorders-5 [33] not including neurodevelopmental or degenerative disorders (for example, not including autism and dementia, but including but not limited to: schizophrenia, depression, anxiety, and personality disorders). A systematic review that included four studies of national survey data found that people with severe mental health conditions such as schizophrenia are significantly more likely to be overweight or obese than other members of the population [32]. One of the studies included in the review found that Australian’s who completed the national survey of psychosis in 2010 were two times more likely to be obese (BMI > 30) compared to the general population in the same time period [34, 35]. Additionally, having a chronic mental health condition, has been independently linked to the development of physical chronic diseases such as cardiovascular disease, [30–32, 36] demonstrating the complexities in addressing the development of chronic disease among this group.

Furthermore, the research literature has also explored the bi-directional impact of physical and mental health. For example, a systematic review and meta-analysis of 26 longitudinal studies among various populations, reported consistent evidence across all populations that smoking cessation was associated with significant reductions in depression, anxiety, stress; and increased psychological well-being and positive affect compared to continued smoking [37]. Systematic review evidence similarly suggests that physical activity reduces anxiety symptoms in both people without a mental health condition, [38] and those with anxiety disorders, [39, 40] depression, [38, 40] schizophrenia, [38, 40] post-traumatic stress disorder, [40] and substance dependence [40]. Further, a meta-analysis of 49 prospective studies found physical activity had a protective effect against the emergence of depression in youths, adults, and elderly persons [41]. Such research highlights the benefits for physical and mental health that can be achieved through addressing health risk behaviours.

In the research literature, the prevalence of inadequate physical activity [28, 29, 42–47] and inadequate fruit and vegetable consumption [28, 29, 45, 46] among people with a mental health condition is consistently reported to be higher than people without a mental health condition, and may be highest for people with mental health conditions requiring inpatient care. This higher prevalence of risk behaviour is demonstrated by comparing the results of three studies conducted in the same geographic region of Australia, utilising comparable methodologies, and consistent definitions of risk behaviours as per national guidelines. Cross-sectional surveys conducted amongst general community health clients (*n* = 1284), community mental health clients (*n* = 558), and psychiatric inpatients (*n* = 2075) revealed a higher prevalence of risk behaviours among community mental health clients compared to general health clients, and further increased risk for psychiatric inpatients for: inadequate fruit and/or vegetable consumption (81% vs 88% vs 95%), and inadequate physical activity (28% vs 47% vs 50%) [48–50].

Internationally and in Australia, guidelines and policies acknowledge the need to provide care to all health service clients who may be at risk for inadequate physical activity and/or fruit and vegetable consumption, [51–53] with additional guidelines and policies existing specifically for clients with a mental health condition [54, 55]. Despite the existence of these guidelines and policies, the reported effectiveness of the provision of preventive care and lifestyle interventions in mental health services to improve such health risk behaviours, [56–60] and the reported interest of mental health service clients in receiving support to improve these behaviours, [49, 61] sub-optimal care for health risk behaviours from general practitioners [62] and community and inpatient mental health services is consistently reported [63]. For instance, a recent a meta-analysis of 26 studies found sub-optimal provision of care (less than 80% of clients in receipt of care) to address inadequate nutrition generally and physical activity across all elements of care (e.g. asking/assessing, advising, assisting, and arranging referral) in community, inpatient, and other mental health services [63]. A number of factors have been implicated as barriers to the provision of preventive care in mental health settings including: clinician attitudes and beliefs about their client’s capability or interest in changing [64, 65]; as well as the risk behaviours of the clinician, where clinicians who are at risk for a particular behaviour themselves may be less likely to provide care to their client for that behaviour [66].

In addition to the existence of policies and guidelines surrounding the type of preventive care to be delivered to people with a mental health condition, guidelines also state the need to include a variety of stakeholders, including clients and their informal family carers, in the planning, development and implementation of mental health policy and practice [67–70]. A family or informal carer is an individual who provides support and assistance without payment to an individual with any physical or mental health condition or disability [71]. Inclusion of multiple stakeholders is recommended in order to deliver a holistic approach to mental health care and increase the effectiveness of health care service treatments and interventions [72–74]. For instance, the New South Wales (NSW) Carer Recognition Act states that carers should be engaged as important stakeholders in the provision of care, including the assessment, planning, delivery, and review of services provided to the person they care for, and should be included in care decision making [75].

The acknowledged role that family carers play in the provision of support to people with a mental health condition, suggests that an alignment between carer expectations regarding what type of care should be provided and the type of care that is actually delivered by services may contribute to service design which may lead to more positive outcomes for people with a mental health condition [76]. The importance of the carer viewpoint is evidenced by the National Mental Health Commission’s 2017 establishment of the Equally Well Implementation Committee, which includes carer members, with the aim of bridging the physical and mental health sectors to ensure holistic care for people with a mental health condition [77].

To date however, there has been limited research undertaken with family carers to understand their expectations regarding what type of care to address the chronic disease risk behaviours should be provided to people with a mental health condition by health and community services. Only two previous studies have focused on this issue relevant to inadequate physical activity and nutrition generally [78–80]. One qualitative study from the US of 13 carers of older adults with serious mental illness found that carers reported a need for guidance from health care professionals regarding strategies to promote weight loss by their family member [78].

The second study, an Australian qualitative study of 31 family carers, reported a desire by carers to be informed about any health service interventions which aimed to address the physical health of the person they cared for so that carers could support such interventions [79]. Additionally, carers reported a desire for services accessed by their family member to provide holistic care, due to their awareness of the bidirectional relationship between physical and mental health. It has been found previously that carers are aware of the bidirectional relationship between physical and mental health, and this has been associated with carer expectations of smoking cessation care provision by services accessed by people with a mental health condition [80]. Further, carers have also reported that their capacity to support their family member in making health behaviour change is impacted by the need to maintain their own physical health and well-being [79]. These two previous studies were limited by small sample sizes, and their lack of focus on physical activity and fruit and vegetable consumption specifically. The absence of any quantitative studies limits understanding of the prevalence of the reported expectations among carers generally.

## Methods

### Aims

Given the limited research concerning carer expectations regarding health service delivery of care addressing client physical activity and nutrition risk behaviours (despite the existence of guidelines and policies championing for the inclusion of the carer perspective) for people with a mental health condition, a study was undertaken to examine: 1) family carers’ expectations of care provision regarding fruit and vegetable consumption and physical activity by health and community services (mental health hospitals, community mental health services, general practice [GP], and non-government organisations [NGOs]) for people with a mental health condition; 2) carers’ own health risk behaviour status and perceptions of the influence of fruit and vegetable consumption and physical activity on mental health; and 3) possible associations of socio-demographic, clinical and attitudinal factors with carer expectations of care provision for fruit and vegetable consumption and physical activity.

### Design and setting

One hundred forty four family carers of adults with a mental illness in one non-metropolitan region in NSW, Australia completed a cross sectional survey from July to November 2013. The study was approved by the Hunter New England Human Research Ethics Committee (No. 13/06/19/5.11) and the University of Newcastle’s Human Research Ethics Committee (No. H-2019-0141; refer to Additional file 1).

### Participants and recruitment

Potential participants were identified through their membership of a non-government carer support organisation that provided free support services, advocacy, training and education to carers of people with a mental health condition [81]. The organisation had operated across the study region for approximately 10 years, in partnership with local mental health services, providing individual and group support. Any member of the public who was a carer of a person with any mental health condition was able to join the organisation without cost as a source of support for their role as a carer. Participants were eligible for the study if they were: 18 years or older and identified themselves as a family carer for someone with any mental health condition who was also over 18 years.

The carer organisation identified potential participants throughout the Hunter New England Local Health District based on members’ previously recorded interest in research participation. The organisation mailed an invitation to participate in the study (Additional file 1), information statement (Additional file 1), survey instrument (Additional file 1) and reply-paid envelope to all such listed members. The invitation letter also included a web link for optional online survey completion. Potential participants who had not responded to the letter after one month were mailed a reminder letter. Most surveys were returned within one month; the remainder were received over a four month period. Additional participants were approached by research team members at carer support group meetings organised by or affiliated with the carer support organisation. Survey completion took approximately 28 min.

### Measures

Carers completed items assessing socio-demographic, clinical and health risk behaviours; which were modified from previous research [82]. Items examining the carer and family member relationship, perceptions regarding the impact of health risk behaviours on mental health, and carer expectations of care provision for health risk behaviours by services were developed with input from mental health staff and carers. Refer to supplementary material for the survey instrument.

### Socio-demographic and clinical characteristics

Six items addressed the age, gender, employment, marital status, highest level of education achieved, and Aboriginal and/or Torres Strait Islander status of both the family carer and person with a mental health condition. Participants were also asked their postcode of residence to determine geographic remoteness and socio-economic index of disadvantage [83, 84].

Participants reported: how many years they had been in a caring role with the person they cared for (years: less than one, 1 to 2, 3 to 10, 11 to 20, more than 20); if they lived in the same residence as that person (yes, no, sometimes); what their relationship was to that person (parent, partner, child, sibling, neighbour, friend, other); and that person’s primary psychiatric diagnosis (schizophrenia, depression, anxiety disorder, panic disorder, bipolar disorder, post-traumatic stress disorder, eating disorder, personality disorder, unsure, other).

### Expectations of care provision

Participants were asked separate questions for each of four types of health care services: mental health hospitals, community mental health services, GP, and/or NGOs. For each, carers were asked if such a service should provide care for a) fruit and vegetable consumption, and b) physical activity for people with a mental health condition (yes, no, unsure). For instance, ‘For someone with a mental health condition, do you think the services below should provide care for fruit and vegetable consumption?’

### Health risk behaviour status

Participants were asked: how many serves of fruit (0, 1, 2 or more, unsure) and vegetables they usually ate each day (0, 1, 2, 3, 4, 5 or more, unsure); and how many days a week they usually did 30 min or more of physical activity (0, 1, 2, 3, 4, 5, 6, 7, unsure, can’t do physical activity for health or treatment reasons).

### Perceived health effects of fruit and vegetable consumption and physical activity

All participants were asked to respond to four items: ‘to what extent do you think eating enough fruit and vegetables can have a positive impact on mental health?’; ‘to what extent do you think doing enough physical activity can have a positive impact on mental health?’; ‘to what extent do you think not eating enough fruit and vegetables can have a negative impact on mental health?’; and ‘to what extent do you think not doing enough physical activity can have a negative impact on mental health?’ (not at all, a little, moderately, very, unsure).

### Data analysis

SPSS version 23 [85] was used to analyse the data. Participant postcode was used to determine the geographic remoteness (major cities, regional, rural) and socio-economic index of disadvantage (disadvantaged, average/advantaged) of the area in which they resided [83, 84]. Response categories for socio-demographic and clinical characteristics were collapsed to two or three categories as shown in Table 1; with the exception of psychiatric diagnosis (four categories). Fruit and vegetable consumption and physical activity levels were dichotomised (adequate vs inadequate) based on the Australian national guidelines, [23, 86] where consuming two or more serves of fruit and five or more serves of vegetables each day was adequate, and participating in at least 30 min of physical activity, at least five days per week was deemed adequate. Items regarding expectations of care provision by the four health and community service settings were condensed to two categories (yes, no or unsure).

**Table 1:** Socio-demographic characteristics

Characteristic	Carer n(%)	Person with a mental health condition n(%)
Age (Years)^1^	18–54: 35 (24.6)	18–34: 58 (40.3)
	55–74: 92 (64.8)	35–54: 67 (46.5)
	75 and over: 15 (10.6)	55 and over: 19 (13.2)
Gender ^1^		
Male	27 (19.0)	96 (66.7)
Employment status		
In the workforce^2^	45 (31.9)	28 (20.3)
Ethnicity ^3^		
Aboriginal and/or Torres Strait Islander origin	5 (3.6)	6 (4.4)
Marital Status ^4^		
Married/ living together in a relationship	105 (73.4)	36 (25.9)
Highest Education Level ^5^		
Less than 4 years high school completed	28 (19.6)	31 (22.6)
4 years high school completed	30 (21.0)	29 (21.2)
More than 4 years high school completed	85 (59.4)	77 (56.2)
Socio-economic index of disadvantage ^1^		
Lowest tertile (Disadvantaged)	78 (54.9)	
Middle/highest tertile (average/advantaged)	64 (45.1)	
Geographic remoteness ^1^		
Major cities (Highly accessible)	44 (31.0)	
Inner regional (Accessible)	77 (54.2)	
Outer regional (Moderately accessible)	21 (14.8)	
Years spent caring for the person with mental illness^1^		
Less than 12 months	5 (3.5)	
1–2 years	11 (7.7)	
3–10 years	47 (33.1)	
11–20 years	37 (26.1)	
More than 20 years	42 (29.6)	
Carer and person with mental illness living in the same residence		
Yes^6^	75 (52.4)	
Carer relationship to person with mental illness^6^		
Parent	88 (61.5)	
Other relation	55 (38.5)	
Psychiatric diagnosis		
Schizophrenia		56 (38.9)
Bipolar disorder		31 (21.5)
Depression		22 (15.3)
Other- single or multiple disorders		35 (24.3)
Fruit consumption^6^		
Inadequate	49 (34.0)	
Vegetable consumption^6^		
Inadequate	98 (68.5)	
Combined fruit and vegetable consumption^6^		
Inadequate	107 (74.8)	
Physical activity ^7^		
Inadequate	76 (57.6)	

^1^ Two missing carer responses

^2^ Three missing carer responses, six missing person with a mental illness responses

^3^ Four missing carer responses, eight missing person with a mental illness responses

^4^One missing carer response, five missing person with a mental illness responses

^5^ One missing carer response, seven missing person with a mental illness responses

^6^ One missing carer response

^7^ Twelve missing carer responses

Descriptive statistics were used to summarise socio-demographic and clinical characteristics, participants’ expectations of care provision by health and community services, risk behaviour status, and perceived effect of fruit and vegetable consumption and physical activity on mental health.

Chi-square analyses using Fisher’s Exact test statistic were used to examine possible associations between all independent variables listed in Table 1 and carers’ perceptions of the impact of the health risk behaviours on mental health with carers’ expectations of care provision for fruit and vegetable consumption and physical activity by each of the four service settings (dependent variables). Independent variables associated at *p* < .25 were subsequently entered into backward stepwise logistic regression models to examine the independent association (*p* < .05) with expectation of care provision (for fruit and vegetable consumption, and physical activity separately) in each of the four service settings (eight models total).

## Results

### Sample characteristics

Of the 371 eligible carers invited to take part; 144 completed the survey (38.8%); 97 by mail, 46 in a carer support group, and 1 online. Participants who completed the survey in a support group were more likely to be 75 years or older (21.7% vs 7.1%, *p* = .005) and to live in a major city (57.8% vs 18.6%, *p* < .001) than participants who completed the survey by post. The majority of participants were female (81.0%), over the age of 54 (75.4%), the parent of the person they cared for (61.5%), and resided with that person (52.4%; Table 1).

### Expectations of care provision

The majority of participants expected all four types of health care services to provide care for fruit and vegetable consumption to people with a mental health condition, with the highest expectation for GPs (81.1%), and the lowest for NGOs (56.2%) (Table 2). The majority of participants also expected all service settings to provide care for physical activity, with the highest expectation for community mental health services (85.9%), and the lowest for NGOs (65.4%).

**Table 2:** Expectations of care in health and community service settings

Item	Response		
	n(%)	n(%)	n(%)
	Yes	No	Unsure
Fruit and Vegetable Consumption Care			
Mental health hospital ^1^	106 (78.5)	17 (12.6)	12 (8.9)
Community mental health service ^2^	102 (76.7)	17 (12.8)	14 (10.5)
General practice ^3^	107 (81.1)	18 (13.6)	7 (5.3)
Non-government organisation ^4^	72 (56.2)	28 (21.9)	28 (21.9)
Physical Activity Care			
Mental health hospital ^2^	110 (82.7)	10 (7.5)	13 (9.8)
Community mental health service ^1^	116 (85.9)	6 (4.4)	13 (9.7)
General practice ^1^	107 (79.2)	14 (10.4)	14 (10.4)
Non-government organisation ^5^	83 (65.4)	13 (10.2)	31 (24.4)

^1^ Nine missing responses

^2^ Eleven missing responses

^3^ Twelve missing responses

^4^ Sixteen missing responses

^5^ Seventeen missing responses

### Health risk behaviour status

The majority of carers were consuming inadequate amounts of vegetables (68.5%), and approximately one third were consuming inadequate amounts of fruit (34.0%). The proportion of carers consuming inadequate fruit or vegetables overall was 74.8%. More than half of the carers reported engaging in inadequate amounts of physical activity (57.6%; Table 1).

### Perceived mental health effects of fruit and vegetable consumption and physical activity

Approximately half the carers perceived that consuming adequate amounts of fruit and vegetables could have a ‘very’ positive impact on mental health (55.9%); 48.6% perceived that inadequate fruit and vegetable consumption would have a ‘very’ negative impact on mental health. Approximately two thirds of participants perceived that adequate physical activity would have a ‘very’ positive impact on mental health (71.3%), and that inadequate physical activity would have a ‘very’ negative impact on mental health (63.8%). Very few participants reported that fruit and vegetable consumption or physical activity would have no impact on mental health (2.1 to 7.0% respectively; Table 3).

**Table 3:** Perceived health effects of fruit and vegetable consumption and physical activity on mental health

Item	Response				
	n(%)	n(%)	n(%)	n(%)	n(%)
	Very	Moderately	A little	Not at all	Unsure
Adequate fruit and vegetable consumption- positive influence on mental health^1^	80 (55.9)	41 (28.7)	11 (7.7)	6 (4.2)	5 (3.5)
Inadequate fruit and vegetable consumption- negative influence on mental health_2_	69 (48.6)	39 (27.5)	13 (9.2)	10 (7.0)	11 (7.7)
Adequate physical activity- positive influence on mental health^1^	102 (71.3)	29 (20.3)	7 (4.9)	3 (2.1)	2 (1.4)
Inadequate physical activity - negative influence on mental health^3^	90 (63.8)	30 (21.3)	6 (4.3)	7 (5.0)	8 (5.6)

^1^ One missing response

^2^ two missing responses

^3^ three missing responses

### Associations between socio-demographic and attitudinal variables, with expectations of care provision

Final regression models are presented in Table 4. Participants perceiving that ‘eating enough fruit and vegetables would have a very positive impact on mental health’ had greater odds of expecting care for fruit and vegetable consumption to be provided in: mental health hospitals (Odds ratio [OR]: 2.61, 95% Confidence Interval [CI]: 1.09–6.26, *p* = .03); GP (OR: 3.30, 95% CI: 1.24–8.51, *p* = .02); and NGOs (OR: 2.30, 95% CI: 1.02–5.17, *p* = .04), compared to carers not holding that view.

**Table 4:** Variables associated with expectations of fruit and vegetable consumption and physical activity care provision

Predictor	OR	95% CI Lower Upper	*p*
FRUIT AND VEGETABLE CONSUMPTION			
Mental health hospitals^1^			
Fruit and vegetable consumption- very positive impact on mental health	2.61	1.09–6.26	0.03*
Community mental health services^2^			
Living in same residence	0.47	0.20–1.12	0.09
Married/de facto carers	0.39	0.13–1.24	0.11
GPs^3^			
Carer inadequate nutrition	0.30	0.08–1.12	0.07
Carer in workforce	3.20	0.98–10.45	0.05
Fruit and vegetable- very positive impact on mental health	3.30	1.24–8.51	0.02*
NGOs^4^			
Carer gender - female	2.32	0.95–5.65	0.07
Fruit and vegetable- very positive impact on mental health	2.30	1.02–5.17	0.04*
Family member psychiatric diagnosis			0.05
Schizophrenia	2.63	0.95–7.27	0.06
Depression	0.83	0.23–2.97	0.77
Bipolar	3.42	1.04–11.18	0.05
Other			Reference
PHYSICAL ACTIVITY			
Mental health hospitals^5^			
Physical activity- very positive impact on mental health	3.70	1.44–9.48	< 0.01*
Community mental health services^6^			
Carer highest education level			< 0.01*
Less than four years high school	0.57	0.15–2.21	0.41
Four years high school	0.15	0.04–0.50	< 0.01*
More than four years high school			Reference
GPs^7^			
Carer highest education level			0.03*
Less than four years high school	0.38	0.13–1.10	0.07
Four years high school	0.28	0.10–0.80	0.02*
More than four years high school			Reference
NGOs^8^			
Carer inadequate physical activity	0.48	0.21–1.10	0.10
Married/de facto carer	0.39	0.14–1.09	0.07

*Significant at *p* < .05

Variables entered intro regression model at *p* < .25:

^1^ Carer gender, carer and family member residential status, impact of fruit and vegetable consumption on mental health

^2^ Carer and family member residential status, carer employment status, carer marital status

^3^ Family member age, carer nutrition risk status, impact of fruit and vegetable consumption on mental health, carer employment status

^4^ Carer age, carer gender, carer nutrition risk status, impact of fruit and vegetable consumption on mental health, carer highest education level, family member psychiatric diagnosis

^5^ Impact of physical activity on mental health, family member highest education level, carer marital status

^6^ Carer highest education level, impact of physical activity on mental health, carer marital status, family member marital status, socio-economic index of disadvantage

^7^ Carer highest education level. Family member highest education level

^8^ Carer age, years in caring relationship, carer physical activity risk status, carer marital status

Carers who perceived ‘doing enough physical activity would have a very positive impact on mental health’ were almost four times more likely to expect care for physical activity in mental health hospitals (OR: 3.70, 95% CI: 1.44–9.84, *p* = <.01), compared to carers not holding that view. Carers who completed four years of high school (School Certificate) had lower odds of expecting physical activity care in community mental health services (OR: .15, 95% CI: .04–.50, *p* = <.01) and GPs (OR: .28, 95% CI: .10–.80, *p* = .02), compared to carers who had completed more than four years high school. There were no other significant associations.

## Discussion

This is the first study to explore the prevalence of family carer expectations regarding care provision for fruit and vegetable consumption and physical activity to people with a mental health condition by a variety of health and community service settings, and factors associated. Many carers expected all four service settings to provide care for fruit and vegetable consumption, (56.2–81.1%) and physical activity (65.4–85.9%). The majority of carers consumed inadequate fruits and vegetables and engaged in inadequate physical activity. The majority of carers perceived that consuming adequate amounts of fruits and vegetables and engaging in adequate amounts of physical activity would have a very positive impact on mental health. Perceptions that the health risk behaviours would have a positive impact on mental health was associated with expectations of care. Carers who held the view that adequate fruit and vegetable consumption would have a very positive impact on mental health were more likely to expect such care in mental health hospitals, GP, and NGOs; whilst carers who believed adequate physical activity would have a very positive impact on mental health were more likely to expect care for that risk behaviour in mental health hospitals. Finally, carers who completed four years of high school were less likely, than carers who completed more than four years of high school, to expect physical activity care provision in community mental health services and GP.

The findings of a high prevalence of carers expecting care to be delivered for both health risk behaviours across all care delivery settings studied align with the recommendations of guidelines and policies regarding the provision of physical health care in all services accessed by people with a mental health condition [51–55]. The results are also consistent with research reporting that people with a mental health condition would find it acceptable to be provided with support to change health risk behaviours from the services they access [28, 49, 61, 87–89]. Such findings highlight the need for health and community services to adhere to existing policies and increase the provision of care for these health risk behaviours to people with a mental health condition given the reported sub-optimal provision of preventive care to date [63].

Findings from implementation research suggest that in order for such care provision to increase in services accessed by people with a mental health condition, further research is required to develop strategies to overcome barriers to the provision of such care. Identified barriers include: a prioritisation of mental health care over health risk behaviours [65, 90]; a lack of confidence in providing preventive care [66, 91]; perceptions of client lack of interest in improving risk behaviours, [90–94] and; a lack of client receptivity to receive behaviour change support [91, 95]; a lack of time to provide such care [96, 97]; lack of training in the provision of care [90, 98]; lack of organisational policies regarding the recording of care provision, [91, 99] and; a lack of reminders to facilitate care provision [97, 100]. Further implementation research is required to identify and understand mental health service characteristics that impede on the provision of preventive care; and subsequently tailor practice change intervention strategies to address such characteristics within mental health services. From the carer perspective, a limited body of literature exists, however it suggests carers do expect holistic care from services accessed by their family members with a mental health condition [79, 101]. Future research could seek to explore expectations of care provision further. It may be that carers might prioritise mental health support over physical health support for their loved one. Some might believe that offering nutrition support would likely limit the time available to deliver more traditional mental health support. Some consumers, staff, and carers may not be aware of the evidence that there are mental health benefits, as well as physical health benefits, from improving physical activity and nutrition condition [38–41].

Additionally, higher expectations of care for both health risk behaviours were reported by carers compared to the proportion of carers who perceived engaging in positive risk behaviours would have a very positive impact on mental health. Further research is required to explore this trend and confirm is such findings are consistent among a larger sample. It may be that expectations of care provision were also influenced by carer knowledge of the positive impact on physical health to be gained from engaging in positive health risk behaviours [79, 102]. Subsequent research is required to explore such a speculation.

A perception that the health risk behaviours have an impact on mental health is consistent with previous research among family carers, where carers reported a perception of a bidirectional relationship between physical and mental health, [79] and have expressed expectations for smoking cessation care to be provided to clients with a mental health condition [80]. Similarities were found between the results of the current study and a previous study exploring carer expectations of smoking cessation care by health and community services [80]. Carers, in a previous study, who perceived that smoking cessation would have a positive impact on mental health were more likely to expect smoking cessation care in mental health hospitals, community mental health services, and non-government organisations [80]. Such findings suggest carers are aware of the impact of health risk behaviours on mental health. Given the prioritisation of mental over physical health care by services, reported by carers [101, 103, 104] and mental health professionals, [105, 106] further dissemination of the bidirectional relationship between physical and mental health [37–41] - and the knowledge of such a link by carers - may aid in increasing physical health care by services catering to clients with a mental health condition. It may be that carers’ knowledge about the link between mental and physical health could be used to advocate for improved preventive care provision in mental health care delivery for their family member.

Carer risk status was not associated with expectations of care for the relevant health risk behaviour in any service studied; similar to a previous study of carer expectations on smoking cessation care by the same service types [80]. Such findings may suggest carers’ own risk status may not impact on their perceptions of physical health care for their family members with a mental health condition. Further research is required to explore this finding to determine if carers may have the potential to support health risk behaviour change among people with a mental health condition (which a limited body of research suggests [78, 79, 101, 102, 107, 108]) and if their own risk status may impact on such a potential. Research among mental health professionals suggests the provision of support to change health risk behaviours may be decreased in those professionals engage in health risk behaviours [66].

The association between carer education level and expectations of physical activity care in community mental health services and GP requires further investigation. It has been found in previous research that individuals with lower levels of education have less knowledge of physical activity guidelines [109]. A recent Australia study (*n* = 615) found that individuals were more likely to be physically active if they were aware of the benefits of physical activity (knowledge increased risk of disease resulting from physical inactivity) [110]. It may be that participating carers with more than four years of high school completed, may have had an increased knowledge of the benefits of physical activity and thus were more likely to expect care within community mental health services.

The results of this research should be considered in the context of the following limitations. This study had a low response rate (< 38%), yielding a small sample (*n* = 144), drawn from members of a carer support organisation within one regional local health district in Australia. Caution should be used when considering the results of the regression analyses given the small sample size. The experiences of participating carers may not be representative of all carers of people with a mental health condition, or of carers from different geographical locations. However, the geographic and socio-economic profile of the participants were largely consistent with characteristics of carers in Australia [71]. Additionally, given participants were recruited based on their previous consent to receive invitations to participate in research; such a recruitment strategy may have resulted in selection bias where these carers who have previously elected to participate in research may not be representative of all carers of people with a mental health condition. The self-reported nature of the data may also be prone to recall and social desirability biases [111]; which would perhaps most likely result in an under-estimation of engagement in chronic disease risk behaviours. Some evidence does suggest however that older adults’ recall of their health behaviours is reliable [112]. Chronic physical conditions were not assessed in the current study. It is unclear if carers’ responses may have differed if their family member with a mental health condition also had a comorbid physical health condition. Future research could aim to assess such comorbidities and the impact on carers’ expectations of support from health services. The current study utilised a broad definition regarding care provision. It is unknown if providing additional details regarding care elements, such as asking about risk behaviours and providing advice, may have impacted the results. Finally, given the cross-sectional nature of the study, no causal inferences can be made. Future research could seek to explore additional factors that could impact on carer’s expectations of care provision in health and community services to people with a mental health condition.

## Conclusions

Given the increasing need to recognise and include family carers as key stakeholders in the provision of care to people with a mental health condition, this research provides novel insight. Findings suggest that many carers are aware of the impact that health risk behaviours can have on mental health and that carers may also expect services accessed by people with a mental health condition to provide care for such risk behaviours. These results reinforce the need for health services to consider providing care for people with a mental health condition that addresses fruit and vegetable consumption and physical activity. Additionally, in line with guidelines recommending the inclusion of client and carer perspectives in service planning, the current findings could be included in discussions regarding the development, provision, and evaluation of new services provided to people with a mental health condition. Future research could explore whether stronger engagement between health services and carers and people with a mental health condition in the development of new services would lead to the implementation of more routine provision of care for chronic disease health risk behaviours.

## Supplementary information

**Additional file 1.** Carers Views. Addressing Physical Health Risk Behaviours

## Data Availability

The dataset used and analysed during the current study is available from the corresponding author on reasonable request.

## Abbreviations

BMI: Body Mass Index
CI: Confidence Interval
DALY: Disability Adjusted Life Years
GP: General Practice
NGO: Non-Government Organisation
NSW: New South Wales
OR: Odds Ratio
