# Supplementary material for: Preventive care for physical activity and fruit and vegetable consumption: a survey of family carer expectations of health service delivery for people with a mental health condition
Source: BMC Health Serv Res. 2020 Mar 12;20:201. doi: 10.1186/s12913-020-5059-0 (PMC7068924; doi:10.1186/s12913-020-5059-0)
Supplement: Supplementary file 1 — Additional file 1. Carers Views. Addressing Physical Health Risk Behaviours [file 12913_2020_5059_MOESM1_ESM.pdf]

Carers Views: Addressing Physical Health Risk Behaviours (version 2, 26/7/13)

Thank you for taking the time to complete this survey.

Please complete the survey only once. As we will be contacting carers via a number of services, it is possible that you could come across the survey a couple of times.

Some of the questions in this survey are about you, and some are about a person you care for who has a mental illness, and who is 18 years of age or older. You should only be completing this if you are aged 18 years or older, and provide care to someone with a mental illness.

If you care for more than one person with a mental illness, we will ask you to select just one person to answer the survey questions about.

Most of the questions in this survey require you to make a response by replacing a cross or a tick in a circle corresponding to your answer.

Not all questions may be relevant to you and you will be instructed to skip certain questions where appropriate. Depending on the answer you give, a skip statement will be alongside and look like this *(Skip question 7, Go to question 8 on page 10).*

1. I consent to completing this survey. I understand that my personal information will remain confidential to the researchers and that the researchers will only gain the information I provide in my responses.
   I understand that the survey is part of a university project and will be conducted as described in the Information Statement, a copy of which I have received.
   I have had the opportunity to have questions answered to my satisfaction.

   Please check the box to indicate your consent.

   ☐

2. Please enter your Identification Number (ID) provided to you by your support organisation. Your Identification Number is attached to a piece of paper attached to this survey. It is a five digit number.
   Please write your ID number here: ☐

We would like to begin by asking some questions **about a person you care for** who has a mental illness, and who is **aged 18 years or older**…

3. How many people with a mental illness, 18 years or older, are you currently caring for?

Please choose **only one** of the following:

- ○ 1 *(Skip question 4, Go to question 5 on page 3)*
- ○ 2
- ○ 3
- ○ 4 or more

For the remaining questions in the survey which ask about the person you care for please **answer questions about only one of the people you care for**.

If you **care for multiple people but only live with one** person over the age of 18 years then **answer all questions about the person that you live with**.

If you live with multiple people with a mental illness that you care for then you can choose which person you want to tell us about as long as they are **over 18 years old**.

4. Can you please tell us why you chose to talk about that person?

You might choose the person you live with, who you spend the most time with, who needs the most care or the person you have been caring for the longest.

It is important that you answer the rest of the questions in this survey about that person only.

Please choose **all** that apply:

- ○ I live with this person
- ○ This person needs the most care
- ○ This person is acutely unwell and cannot care for themselves
- ○ I spend the most time with this person
- ○ I have been caring for this person the longest
- ○ I am closest with this person
- ○ I receive a carer pension for this person
- ○ I spend more of my energy on this person than the other people I care for
- ○ Other: Please describe

For the following questions about the person you care for, please answer in terms of your 'usual' relationship with them.

For example, if the person you care for usually lives with you but is in an inpatient facility at the current time, please answer questions based on what your behaviours are like when you are living together.

5. How many years have you been caring for this person?
   Please choose **only one** of the following:
   - ○ Less than one year
   - ○ 1-2 years
   - ○ 3-10 years
   - ○ 11-20 years
   - ○ more than 20 years

6. Are you living in the same residence as the person you provide care for?
   Please choose **only one** of the following:
   - ○ Yes
   - ○ No
   - ○ Sometimes

7. What is your relationship to the person you are providing care for?
   What are you to them? For example, if you are their mother select 'parent'.
   Please choose **only one** of the following:

   I am their:
   - ○ Parent
   - ○ Partner
   - ○ Child
   - ○ Sibling (brother or sister)
   - ○ Neighbour
   - ○ Friend
   - ○ Other: Please describe

8. How many days a week would you usually spend time with the person you care for?

9. Are you the sole carer of this person?
Please choose **only one** of the following:

- ○ Yes *(skip question 10. Go to question 11 on this page)*
- ○ No

10. If no, who else cares for this person?
Please choose **all** that apply:

- ○ My spouse/ partner
- ○ Another relative of the person being cared for
- ○ Neighbour
- ○ Friend of mine
- ○ Friend of the person being cared for
- ○ Other

11. What is the age of **the person you care for**?

12. What is **their** gender?

- ○ Female
- ○ Male

13. What is the primary psychiatric diagnosis **of the person you care for**?
Please choose **only one** of the following:

- ○ Schizophrenia
- ○ Depression
- ○ Anxiety disorder
- ○ Panic disorder
- ○ Bipolar disorder
- ○ Post-traumatic stress disorder
- ○ Eating disorder
- ○ Personality disorder
- ○ Dementia
- ○ Unsure
- ○ Other: Please describe

14. What is **their** current employment status?

- ○ Employed full time
- ○ Employed part time or casual
- ○ Not currently employed- but seeking employment
- ○ Not currently employed- not seeking employment

15. Are **they** of Aboriginal or Torres Strait Islander origin?

- ○ Yes, Aboriginal origin
- ○ Yes, Torres Strait Islander origin
- ○ Yes, both Aboriginal and Torres Strait Islander origin
- ○ No

16. What is **their** present marital status?

- ○ Never married
- ○ Married or living together in a relationship
- ○ Divorced/ Separated
- ○ Widowed

17. What is the highest level of education **they** have achieved?

- ○ No formal schooling or attended primary school only
- ○ Some high school with less than four years completed
- ○ School certificate, Intermediate, Year 10, 4th Form
- ○ Completed HSC, Leaving, Year 12 or 6th Form
- ○ TAFE certificate or diploma
- ○ University, College of Advanced Education, Degree or higher

The questions in this next section are about **the physical health behaviours of the person you care for**.

The questions are about smoking, fruit and vegetable consumption, alcohol consumption and physical activity.

Please answer these questions about the person you care for.

18. How many serves of vegetables does the person you care for usually eat each day?

One serve of vegetables or legumes is equal to: ½ cup green leafy vegetables like cabbage, spinach, Brussels sprouts or cauliflower; ½ cup green beans, zucchini, mushrooms, turnips, swede or eggplant; 1 cup salad vegetables such as tomato, capsicum and celery; 1 medium sized potato or parsnip.

Please choose **only one** of the following:

- ○ 0
- ○ 1
- ○ 2
- ○ 3
- ○ 4
- ○ 5 or more
- ○ Unsure

19. How many serves of fruit do they usually eat each day?

One serve of fruit is equal to: 150 grams (5.29 oz.) of fresh fruit or; one medium sized fruit (e.g. apple); 2 smaller pieces (e.g. apricots); 1 cup canned or chopped fruit; ½ cup/ 125 ml (5 fl oz.) 100% fruit juice; 1.5 tablespoon dried fruit (e.g. sultanas or 4 dried apricot halves).

Please choose **only one** of the following:

- ○ 0
- ○ 1
- ○ 2 or more
- ○ Unsure

20. How many days a week does the person you care for usually do 30 minutes or more of physical activity?

By physical activity we mean any activity that increases your heart rate or makes you breathe harder than normal. This can include brisk walking, swimming, team sports or even things like gardening. You can add up your total time during the day, for example walking to the shops and back.

Please choose **only one** of the following:

- ○ 0
- ○ 1
- ○ 2
- ○ 3
- ○ 4
- ○ 5
- ○ 6
- ○ 7, everyday
- ○ Unsure
- ○ Can't do physical activity for health or treatment reasons

21. Do they live in a smoke-free household?

By this we mean people may be smokers but no smoking is permitted inside the residence.

Please choose **only one** of the following:

- ○ Yes
- ○ No
- ○ Unsure

22. Is the person you care for a smoker of any tobacco products?
This could include 'roll your own', cigars, pipe etc.
Please choose **only one** of the following:

- ○ Yes, daily *(go to the next question)*
- ○ Yes, at least once a week *(go to the next question)*
- ○ Yes, less than once a week *(go to the next question)*
- ○ No, quit within the last 4 months *(go to question 30 on page 10)*
- ○ No, quit longer than 4 months ago *(go to question 30 on page 10)*
- ○ No, never smoked *(go to question 31 on page 10)*

23. How many cigarettes (or cigars or pipes) are they usually smoking each day?
Please choose **only one** of the following:

- ○ 10 or less
- ○ 11 to 20
- ○ 21 to 30
- ○ 31 or more
- ○ Unsure

24. How soon after waking are they usually having their first smoke?
Please choose **only one** of the following:

- ○ Within 5 minutes
- ○ 6 to 30 minutes
- ○ 31 to 60 minutes
- ○ More than 60 minutes
- ○ Unsure

25. In the last year, did they ever on purpose quit smoking for at least 24 hours?
Please choose **only one** of the following:

- ○ Yes
- ○ No
- ○ Unsure

26. How many times have they ever made an attempt to quit smoking in the past?
Please choose **only one** of the following:

- ○ Never *(Skip question 27. Go to question 28 on this page)*
- ○ Once
- ○ 2 to 4 attempts
- ○ 5 or more attempts
- ○ Unsure *(Skip question 27. Go to question 28 on this page)*

27. How long ago was their last quit attempt?
Please choose **only one** of the following:

- ○ Currently trying to quit
- ○ 3 months or less
- ○ Between 3 and 12 months ago
- ○ More than one year ago
- ○ Unsure

28. Do they plan to quit smoking?
Please choose **only one** of the following:

- ○ Yes
- ○ No *(Skip question 29. Go to question 30 on page 10)*
- ○ Unsure *(Skip question 29. Go to question 30 on page 10)*

29. When do they plan to quit smoking?
Please choose **only one** of the following:

- ○ Within the next month
- ○ Within the next 2 to 6 months
- ○ More than 6 months
- ○ Unsure

30. Which of the following have they ever used in an attempt to quit smoking?
Please choose **all** that apply:

- ○ Nicotine Replacement Therapy (NRT); such as the patches or the chewing gum
- ○ E cigarette
- ○ Quitline
- ○ GP (doctor) advice
- ○ Hypnosis
- ○ Acupuncture
- ○ Champix (Varenicline)
- ○ Zyban (Buproprion)
- ○ Other medications
- ○ Cold turkey (they just stopped on their own with no assistance)
- ○ None of these

31. How often does the person you care for have a drink containing alcohol?
Please choose **only one** of the following:

- ○ Never, not drinking alcohol *(go to question 34 on page 13)*
- ○ Monthly or less
- ○ 2 to 4 times a month
- ○ 2 to 3 times a week
- ○ 4 or more times a week
- ○ Unsure *(go to question 34 on page 13)*

32. How many standard drinks would they have on a typical drinking day?
Please refer to the diagrams on the following pages as a guide of standard drink measurements.
Please choose **only one** of the following:

- ○ 1 to 2
- ○ 3 to 4
- ○ 5 to 6
- ○ 7 to 9
- ○ 10 or more
- ○ Unsure

NUMBER OF STANDARD DRINKS – BEER

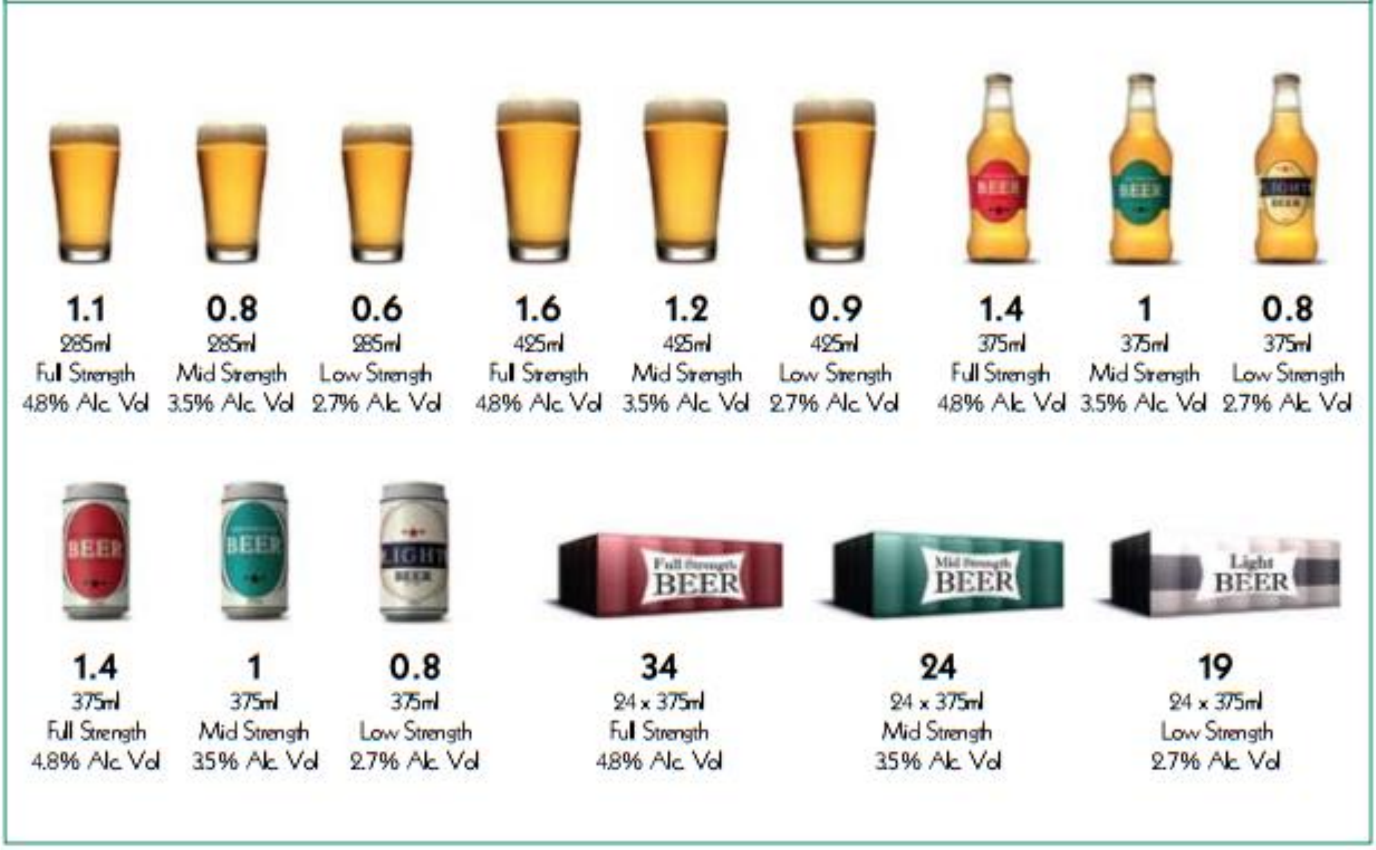

NUMBER OF STANDARD DRINKS – WINE

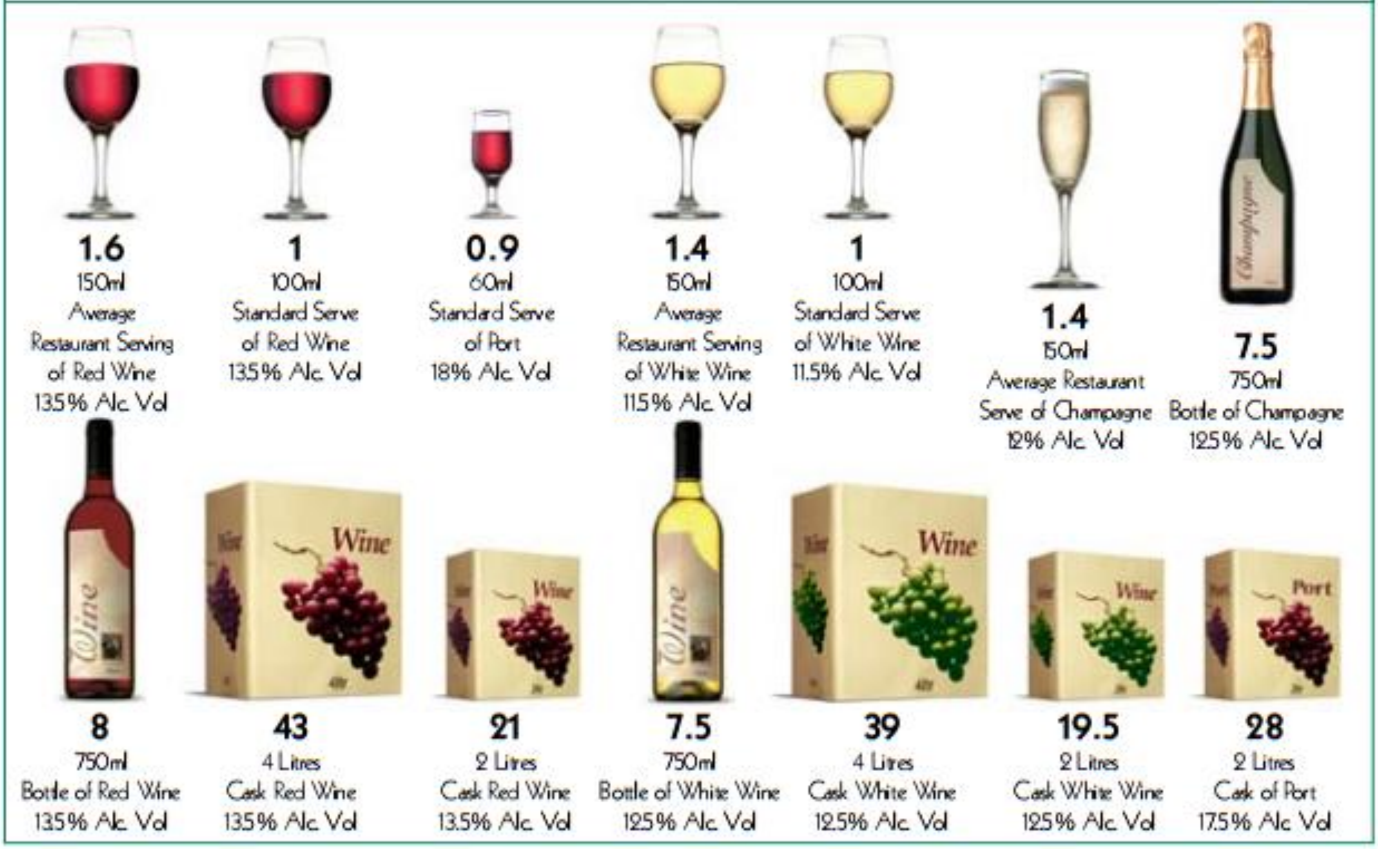

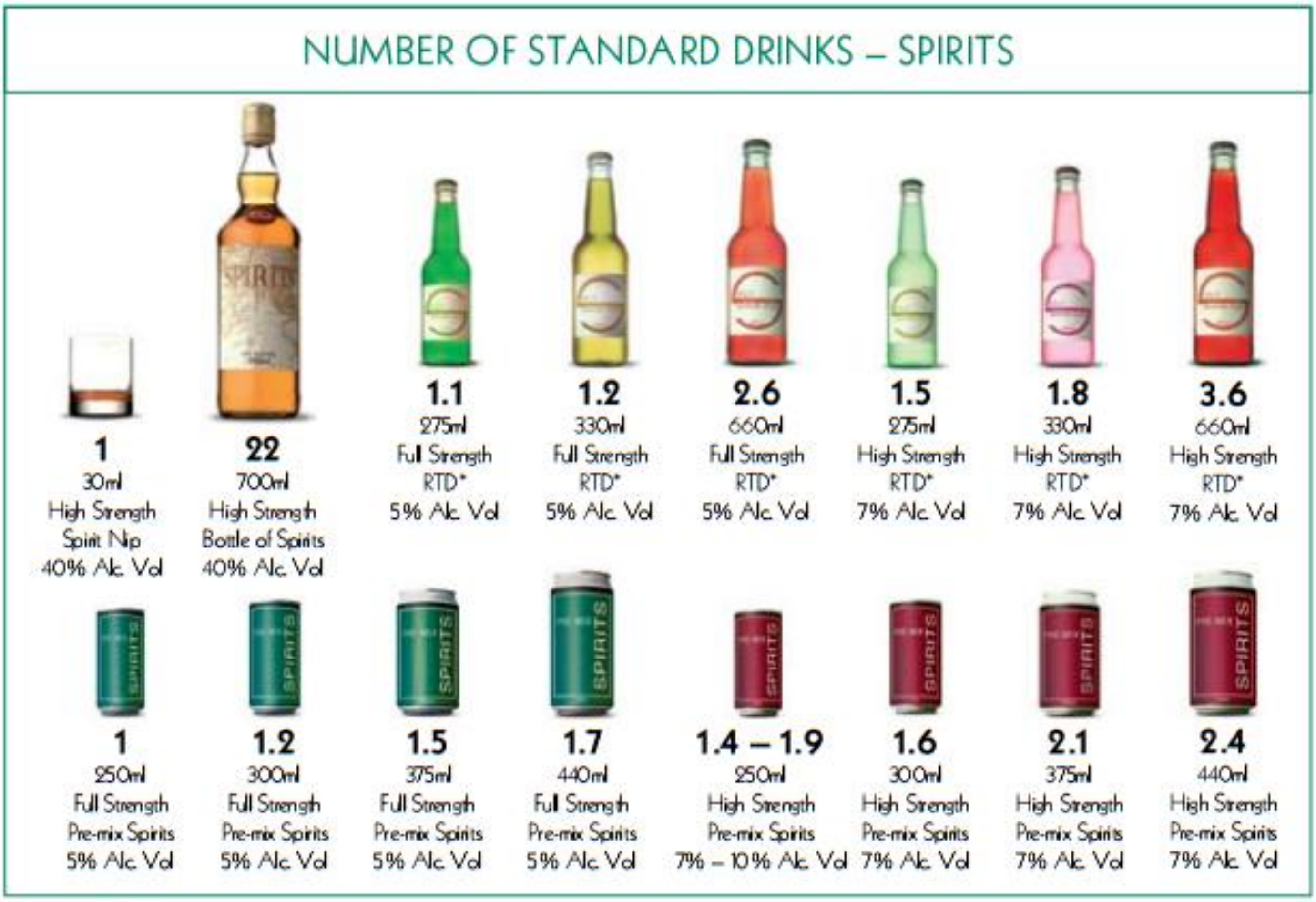

* Ready-to-Drink

33. How often would they have four or more standard drinks on one occasion?
Please choose **only one** of the following:

- ○ Never
- ○ Less than monthly
- ○ Monthly
- ○ Weekly
- ○ Daily or almost daily
- ○ Unsure

34. In the last year, has the person you care for ever expressed an interest in improving any of the following health behaviours?
Please choose the appropriate response for **each** item:
<u>You may want to answer Not Applicable for "Alcohol" or "Smoking" if the person you care for is not a smoker or does not drink alcohol.</u>

	**Yes**	**No**	**Unsure**	**Not Applicable, not a smoker or not drinking alcohol**
Fruit and vegetable consumption	○	○	○	○
Physical activity	○	○	○	○
Alcohol	○	○	○	○
Smoking	○	○	○	○

35. Do you think any of the following factors are health risks for the person you care for?
Please choose the appropriate response for **each** item:

	**Yes**	**No**	**Unsure**	**Not Applicable, not a smoker or not drinking alcohol**
Not eating enough fruit and vegetables	○	○	○	○
Not doing enough physical activity	○	○	○	○
Alcohol use	○	○	○	○
Smoking	○	○	○	○

Mental Health Services

The next few questions are about whether mental health services should provide care for physical health behaviours for people with mental illness.

They are important for everyone to answer.

We would like you to answer the following questions even if the person you care for has not visited that type of service.

Please provide one answer for each service listed in each question.

36. For someone with a mental illness, do you think the services below should provide care for **smoking**?

	Yes	No	Unsure
Hunter New England mental health hospital/ unit (e.g. The Mater; Banksia)	◯	◯	◯
Hunter New England community mental health service/ team (e.g. Newcastle Mental Health Services)	◯	◯	◯
Doctor/ General Practitioner (GP)	◯	◯	◯
Non-Government Organisation (NGO) (e.g. NEAMI, Richmond PRA)	◯	◯	◯
Psychiatrist in private practice	◯	◯	◯
Psychologist in private practice	◯	◯	◯
Community drug and alcohol service	◯	◯	◯
General hospital emergency service	◯	◯	◯
Private mental health hospital/ unit (e.g. Warners Bay Private Hospital, Lake Side Clinic)	◯	◯	◯

37. For someone with a mental illness, do you think the services below should provide care for **fruit and vegetable consumption**?

	Yes	No	Unsure
Hunter New England mental health hospital/ unit	○	○	○
Hunter New England community mental health service/ team	○	○	○
Doctor/ General Practitioner (GP)	○	○	○
Non-Government Organisation (NGO)	○	○	○
Psychiatrist in private practice	○	○	○
Psychologist in private practice	○	○	○
Community drug and alcohol service	○	○	○
General hospital emergency service	○	○	○
Private mental health hospital/ unit	○	○	○

38. For someone with a mental illness, do you think the services below should provide care for **alcohol**?

	Yes	No	Unsure
Hunter New England mental health hospital/ unit	○	○	○
Hunter New England community mental health service/ team	○	○	○
Doctor/ General Practitioner (GP)	○	○	○
Non-Government Organisation (NGO)	○	○	○
Psychiatrist in private practice	○	○	○
Psychologist in private practice	○	○	○
Community drug and alcohol service	○	○	○
General hospital emergency service	○	○	○
Private mental health hospital/ unit	○	○	○

39. For someone with a mental illness, do you think the services below should provide care for **physical activity**?

	Yes	No	Unsure
Hunter New England mental health hospital/ unit	◯	◯	◯
Hunter New England community mental health service/ team	◯	◯	◯
Doctor/ General Practitioner (GP)	◯	◯	◯
Non-Government Organisation (NGO)	◯	◯	◯
Psychiatrist in private practice	◯	◯	◯
Psychologist in private practice	◯	◯	◯
Community drug and alcohol service	◯	◯	◯
General hospital emergency service	◯	◯	◯
Private mental health hospital/ unit	◯	◯	◯

This set of questions is about mental health services that **the person you care for** may have visited. We want to know if the physical health behaviours of the person you care for have been discussed through these services. If the person you care for hasn't used a particular type of service, then you will be directed to skip the remaining questions about that type of service, and go to the next type of service.

Care received from a **Hunter New England mental health hospital or inpatient unit** e.g. the Mater; Banksia.

40. When was the most recent time that the person you provide care for attended (received care from) **a Hunter New England mental health hospital/ unit**?

- ○ Within the last year
- ○ More than 1 year ago
- ○ Never *(Go to question 49, on page 19)*
- ○ Unsure *(Go to question 49, on page 19)*

41. Has the person you care for ever been asked, when attending a Hunter New England mental health hospital/ unit, if they **smoke**?

- ○ Yes
- ○ No *(Go to question 43, on this page)*
- ○ Unsure *(Go to question 43, on this page)*

42. And if so, were they provided with any advice, treatment or referral relating to smoking?

- ○ Yes
- ○ No
- ○ Unsure

43. Has the person you care for ever been asked, when attending a Hunter New England mental health hospital/ unit, about their **fruit and vegetable consumption**?

- ○ Yes
- ○ No *(Go to question 45, on page 18)*
- ○ Unsure *(Go to question 45, on page 18)*

44. And if so, were they provided with any advice, treatment or referral relating to their fruit and vegetable consumption?

- ○ Yes
- ○ No
- ○ Unsure

45. Has the person you care for ever been asked, when attending a Hunter New England mental health hospital/ unit, if they **drink alcohol**?

- ○ Yes
- ○ No *(Go to question 47, on this page)*
- ○ Unsure *(Go to question 47, on this page)*

46. And if so, were they provided with any advice, treatment or referral relating to their alcohol use?

- ○ Yes
- ○ No
- ○ Unsure

47. Has the person you care for ever been asked, when attending a Hunter New England mental health hospital/ unit about their **physical activity levels**?

- ○ Yes
- ○ No *(Go to question 49, on page 19)*
- ○ Unsure *(Go to question 49, on page 19)*

48. And if so, were they provided with any advice, treatment or referral relating to their physical activity levels?

- ○ Yes
- ○ No
- ○ Unsure

Care received from a **Hunter New England community mental health service/ team** e.g. Newcastle Mental Health Services, Lake Macquarie Mental health services.

49. When was the most recent time that the person you provide care for attended (received care from) **a Hunter New England community mental health service/ team**?

- ○ Within the last year
- ○ More than 1 year ago
- ○ Never *(Go to question 58, on page 21)*
- ○ Unsure *(Go to question 58, on page 21)*

50. Has the person you care for ever been asked, when attending a Hunter New England community mental health service/ team, if they **smoke**?

- ○ Yes
- ○ No *(Go to question 52, on this page)*
- ○ Unsure *(Go to question 52, on this page)*

51. And if so, were they provided with any advice, treatment or referral relating to smoking?

- ○ Yes
- ○ No
- ○ Unsure

52. Has the person you care for ever been asked, when attending a Hunter New England community mental health service/ team, about their **fruit and vegetable consumption**?

- ○ Yes
- ○ No *(Go to question 54, on page 20)*
- ○ Unsure *(Go to question 54, on page 20)*

53. And if so, were they provided with any advice, treatment or referral relating to their fruit and vegetable consumption?

- ○ Yes
- ○ No
- ○ Unsure

54. Has the person you care for ever been asked, when attending a Hunter New England community mental health service/ team, if they **drink alcohol**?

- ○ Yes
- ○ No *(Go to question 56, on this page)*
- ○ Unsure *(Go to question 56, on this page)*

55. And if so, were they provided with any advice, treatment or referral relating to their alcohol use?

- ○ Yes
- ○ No
- ○ Unsure

56. Has the person you care for ever been asked, when attending a Hunter New England community mental health service/ team, about their **physical activity levels**?

- ○ Yes
- ○ No *(Go to question 58, on page 21)*
- ○ Unsure *(Go to question 58, on page 21)*

57. And if so, were they provided with any advice, treatment or referral relating to their physical activity levels?

- ○ Yes
- ○ No
- ○ Unsure

Care received from a **Doctor/ General Practitioner (GP)**.

58. When was the most recent time that the person you provide care for attended (received care from) **a doctor/ GP**?

- ○ Within the last year
- ○ More than 1 year ago
- ○ Never *(Go to question 67, on page 23)*
- ○ Unsure *(Go to question 67, on page 23)*

59. Has the person you care for ever been asked, when attending a doctor/ GP, if they **smoke**?

- ○ Yes
- ○ No *(Go to question 61, on this page)*
- ○ Unsure *(Go to question 61, on this page)*

60. And if so, were they provided with any advice, treatment or referral relating to smoking?

- ○ Yes
- ○ No
- ○ Unsure

61. Has the person you care for ever been asked, when attending a doctor/ GP, about their **fruit and vegetable consumption**?

- ○ Yes
- ○ No *(Go to question 63, on page 22)*
- ○ Unsure *(Go to question 63, on page 22)*

62. And if so, were they provided with any advice, treatment or referral relating to their fruit and vegetable consumption?

- ○ Yes
- ○ No
- ○ Unsure

63. Has the person you care for ever been asked, when attending a doctor/ GP, if they **drink alcohol**?

- ○ Yes
- ○ No *(Go to question 65, on this page)*
- ○ Unsure *(Go to question 65, on this page)*

64. And if so, were they provided with any advice, treatment or referral relating to their alcohol use?

- ○ Yes
- ○ No
- ○ Unsure

65. Has the person you care for ever been asked, when attending a doctor/ GP, about their **physical activity levels**?

- ○ Yes
- ○ No *(Go to question 67, on page 23)*
- ○ Unsure *(Go to question 67, on page 23)*

66. And if so, were they provided with any advice, treatment or referral relating to their physical activity levels?

- ○ Yes
- ○ No
- ○ Unsure

Care received from a **Non-Government Organisation (NGO)** e.g. NEAMI, Richmond PRA.

67. When was the most recent time that the person you provide care for attended (received care from) **a NGO**?

 ○ Within the last year

 ○ More than 1 year ago

 ○ Never *(Go to question 76, on page 25)*

 ○ Unsure *(Go to question 76, on page 25)*

68. Has the person you care for ever been asked, when attending a NGO, if they **smoke**?

 ○ Yes

 ○ No *(Go to question 70, on this page)*

 ○ Unsure *(Go to question 70, on this page)*

69. And if so, were they provided with any advice, treatment or referral relating to smoking?

 ○ Yes

 ○ No

 ○ Unsure

70. Has the person you care for ever been asked, when attending a NGO, about their **fruit and vegetable consumption**?

 ○ Yes

 ○ No *(Go to question 72, on page 24)*

 ○ Unsure *(Go to question 72, on page 24)*

71. And if so, were they provided with any advice, treatment or referral relating to their fruit and vegetable consumption?

- ○ Yes
- ○ No
- ○ Unsure

72. Has the person you care for ever been asked, when attending a NGO, if they **drink alcohol**?

- ○ Yes
- ○ No *(Go to question 74, on this page)*
- ○ Unsure *(Go to question 74, on this page)*

73. And if so, were they provided with any advice, treatment or referral relating to their alcohol use?

- ○ Yes
- ○ No
- ○ Unsure

74. Has the person you care for ever been asked, when attending a NGO, about their **physical activity levels**?

- ○ Yes
- ○ No *(Go to question 76, on page 25)*
- ○ Unsure *(Go to question 76, on page 25)*

75. And if so, were they provided with any advice, treatment or referral relating to their physical activity levels?

- ○ Yes
- ○ No
- ○ Unsure

Care received from a **psychiatrist in private practice**.

76. When was the most recent time that the person you provide care for attended (received care from) **a psychiatrist in private practice**?

   - ○ Within the last year
   - ○ More than 1 year ago
   - ○ Never *(Go to question 85, on page 27)*
   - ○ Unsure *(Go to question 85, on page 27)*

77. Has the person you care for ever been asked, when attending a psychiatrist in private practice, if they **smoke**?

   - ○ Yes
   - ○ No *(Go to question 79, on this page)*
   - ○ Unsure *(Go to question 79, on this page)*

78. And if so, were they provided with any advice, treatment or referral relating to smoking?

   - ○ Yes
   - ○ No
   - ○ Unsure

79. Has the person you care for ever been asked, when attending a psychiatrist in private practice, about their **fruit and vegetable consumption**?

   - ○ Yes
   - ○ No *(Go to question 81, on page 26)*
   - ○ Unsure *(Go to question 81, on page 26)*

80. And if so, were they provided with any advice, treatment or referral relating to their fruit and vegetable consumption?

- ○ Yes
- ○ No
- ○ Unsure

81. Has the person you care for ever been asked, when attending a psychiatrist in private practice, if they **drink alcohol**?

- ○ Yes
- ○ No *(Go to question 83, on this page)*
- ○ Unsure *(Go to question 83, on this page)*

82. And if so, were they provided with any advice, treatment or referral relating to their alcohol use?

- ○ Yes
- ○ No
- ○ Unsure

83. Has the person you care for ever been asked, when attending a psychiatrist in private practice, about their **physical activity levels**?

- ○ Yes
- ○ No *(Go to question 85, on page 27)*
- ○ Unsure *(Go to question 85, on page 27)*

84. And if so, were they provided with any advice, treatment or referral relating to their physical activity levels?

- ○ Yes
- ○ No
- ○ Unsure

Care received from a **psychologist in private practice**.

85. When was the most recent time that the person you provide care for attended (received care from) **a psychologist in private practice**?

   - ○ Within the last year
   - ○ More than 1 year ago
   - ○ Never *(Go to question 94, on page 29)*
   - ○ Unsure *(Go to question 94, on page 29)*

86. Has the person you care for ever been asked, when attending a psychologist in private practice, if they **smoke**?

   - ○ Yes
   - ○ No *(Go to question 88, on this page)*
   - ○ Unsure *(Go to question 88, on this page)*

87. And if so, were they provided with any advice, treatment or referral relating to smoking?

   - ○ Yes
   - ○ No
   - ○ Unsure

88. Has the person you care for ever been asked, when attending a psychologist in private practice, about their **fruit and vegetable consumption**?

   - ○ Yes
   - ○ No *(Go to question 90, on page 28)*
   - ○ Unsure *(Go to question 90, on page 28)*

89. And if so, were they provided with any advice, treatment or referral relating to their fruit and vegetable consumption?

- ◯ Yes
- ◯ No
- ◯ Unsure

90. Has the person you care for ever been asked, when attending a psychologist in private practice, if they **drink alcohol**?

- ◯ Yes
- ◯ No *(Go to question 92, on this page)*
- ◯ Unsure *(Go to question 92, on this page)*

91. And if so, were they provided with any advice, treatment or referral relating to their alcohol use?

- ◯ Yes
- ◯ No
- ◯ Unsure

92. Has the person you care for ever been asked, when attending a psychologist in private practice, about their **physical activity levels**?

- ◯ Yes
- ◯ No *(Go to question 94, on page 29)*
- ◯ Unsure *(Go to question 94, on page 29)*

93. And if so, were they provided with any advice, treatment or referral relating to their physical activity levels?

- ◯ Yes
- ◯ No
- ◯ Unsure

Care received from a **community drug and alcohol service**.

94. When was the most recent time that the person you provide care for attended (received care from) **a community drug and alcohol service**?

- ○ Within the last year
- ○ More than 1 year ago
- ○ Never *(Go to question 103, on page 31)*
- ○ Unsure *(Go to question 103, on page 31)*

95. Has the person you care for ever been asked, when attending a community drug and alcohol service, if they **smoke**?

- ○ Yes
- ○ No *(Go to question 97, on this page)*
- ○ Unsure *(Go to question 97, on this page)*

96. And if so, were they provided with any advice, treatment or referral relating to smoking?

- ○ Yes
- ○ No
- ○ Unsure

97. Has the person you care for ever been asked, when attending a community drug and alcohol service, about their **fruit and vegetable consumption**?

- ○ Yes
- ○ No *(Go to question 99, on page 30)*
- ○ Unsure *(Go to question 99, on page 30)*

98. And if so, were they provided with any advice, treatment or referral relating to their fruit and vegetable consumption?

- ○ Yes
- ○ No
- ○ Unsure

99. Has the person you care for ever been asked, when attending a community drug and alcohol service, if they **drink alcohol**?

- ○ Yes
- ○ No *(Go to question 101, on this page)*
- ○ Unsure *(Go to question 101, on this page)*

100. And if so, were they provided with any advice, treatment or referral relating to their alcohol use?

- ○ Yes
- ○ No
- ○ Unsure

101. Has the person you care for ever been asked, when attending a community drug and alcohol service, about their **physical activity levels**?

- ○ Yes
- ○ No *(Go to question 103, on page 31)*
- ○ Unsure *(Go to question 103, on page 31)*

102. And if so, were they provided with any advice, treatment or referral relating to their physical activity levels?

- ○ Yes
- ○ No
- ○ Unsure

Care received from a **general hospital emergency service**.

103. When was the most recent time that the person you provide care for attended (received care from) **a general hospital emergency service**?

- ○ Within the last year
- ○ More than 1 year ago
- ○ Never *(Go to question 112, on page 33)*
- ○ Unsure *(Go to question 112, on page 33)*

104. Has the person you care for ever been asked, when attending a general hospital emergency service, if they **smoke**?

- ○ Yes
- ○ No *(Go to question 106, on this page)*
- ○ Unsure *(Go to question 106, on this page)*

105. And if so, were they provided with any advice, treatment or referral relating to smoking?

- ○ Yes
- ○ No
- ○ Unsure

106. Has the person you care for ever been asked, when attending a general hospital emergency service, about their **fruit and vegetable consumption**?

- ○ Yes
- ○ No *(Go to question 108, on page 32)*
- ○ Unsure *(Go to question 108, on page 32)*

107. And if so, were they provided with any advice, treatment or referral relating to their fruit and vegetable consumption?

- ○ Yes
- ○ No
- ○ Unsure

108. Has the person you care for ever been asked, when attending a general hospital emergency service, if they **drink alcohol**?

- ○ Yes
- ○ No *(Go to question 110, on this page)*
- ○ Unsure *(Go to question 110, on this page)*

109. And if so, were they provided with any advice, treatment or referral relating to their alcohol use?

- ○ Yes
- ○ No
- ○ Unsure

110. Has the person you care for ever been asked, when attending a general hospital emergency service, about their **physical activity levels**?

- ○ Yes
- ○ No *(Go to question 112, on page 33)*
- ○ Unsure *(Go to question 112, on page 33)*

111. And if so, were they provided with any advice, treatment or referral relating to their physical activity levels?

- ○ Yes
- ○ No
- ○ Unsure

Care received from a **private mental health hospital/ unit e.g.** Warners Bay Private Hospital, Lake Side Clinic.

112. When was the most recent time that the person you provide care for attended (received care from) **a private mental health hospital**?

- ○ Within the last year
- ○ More than 1 year ago
- ○ Never *(Go to question 121, on page 35)*
- ○ Unsure *(Go to question 121, on page 35)*

113. Has the person you care for ever been asked, when attending a private mental health hospital, if they **smoke**?

- ○ Yes
- ○ No *(Go to question 115, on this page)*
- ○ Unsure *(Go to question 115, on this page)*

114. And if so, were they provided with any advice, treatment or referral relating to smoking?

- ○ Yes
- ○ No
- ○ Unsure

115. Has the person you care for ever been asked, when attending a private mental health hospital, about their **fruit and vegetable consumption**?

- ○ Yes
- ○ No *(Go to question 117, on page 34)*
- ○ Unsure *(Go to question 117, on page 34)*

116. And if so, were they provided with any advice, treatment or referral relating to their fruit and vegetable consumption?

- ○ Yes
- ○ No
- ○ Unsure

117. Has the person you care for ever been asked, when attending a private mental health hospital, if they **drink alcohol**?

- ○ Yes
- ○ No *(Go to question 119, on this page)*
- ○ Unsure *(Go to question 119, on this page)*

118. And if so, were they provided with any advice, treatment or referral relating to their alcohol use?

- ○ Yes
- ○ No
- ○ Unsure

119. Has the person you care for ever been asked, when attending a private mental health hospital about their **physical activity levels**?

- ○ Yes
- ○ No *(Go to question 121, on page 35)*
- ○ Unsure *(Go to question 121, on page 35)*

120. And if so, were they provided with any advice, treatment or referral relating to their physical activity levels?

- ○ Yes
- ○ No
- ○ Unsure

These two questions are about your view of whether health behaviours influence mental health.

In general, do you think health behaviours can influence mental health?

121. To what extent do you think health behaviours can have a **positive impact** on mental health?
Please choose the appropriate response for each item:

	Not at all	A little	Moderately	Very	Unsure
Eating enough fruit and vegetables	○	○	○	○	○
Doing enough physical activity	○	○	○	○	○
Decreasing alcohol use	○	○	○	○	○
Quitting smoking	○	○	○	○	○

122. To what extent do you think health behaviours can have a **negative impact** on mental health?
Please choose the appropriate response for each item:

	Not at all	A little	Moderately	Very	Unsure
Not eating enough fruit and vegetables	○	○	○	○	○
Not doing enough physical activity	○	○	○	○	○
Using too much alcohol	○	○	○	○	○
Smoking	○	○	○	○	○

Carer Role

This set of questions is about **your role as a carer**.

123. How important do you feel it is for you to try and have a positive influence on the health behaviours of the person you care for?
Please choose the appropriate response for each item:
You may want to answer Not Applicable for "Alcohol" or "Smoking" if the person you care for is not a smoker or does not drink alcohol.

	**Not at all important**	**A little important**	**Somewhat important**	**Very important**	**Unsure**	**Not applicable, not a smoker or not drinking alcohol**
Fruit and vegetable consumption	◯	◯	◯	◯	◯	◯
Physical activity	◯	◯	◯	◯	◯	◯
Alcohol	◯	◯	◯	◯	◯	◯
Smoking	◯	◯	◯	◯	◯	◯

124. To what extent do you currently try to have a **positive influence** on the health behaviours of the person you care for?
Please choose the appropriate response for each item:

	**I don't try**	**I try to address their behaviours sometimes**	**I try to address their behaviours most of the time**	**I try to address their behaviour all the time**	**Unsure**	**Not applicable, not a smoker or not drinking alcohol**
Fruit and vegetable consumption	◯	◯	◯	◯	◯	◯
Physical activity	◯	◯	◯	◯	◯	◯
Alcohol	◯	◯	◯	◯	◯	◯
Smoking	◯	◯	◯	◯	◯	◯

125. To what extent do you think it's possible for you to have a positive influence on the health behaviours of the person you care for?
Please choose the appropriate response for each item:

	**Not at all possible**	**Sometimes possible**	**Often possible**	**Always possible**	**Unsure**	**Not applicable, not a smoker or not drinking alcohol**
Fruit and vegetable consumption	◯	◯	◯	◯	◯	◯
Physical activity	◯	◯	◯	◯	◯	◯
Alcohol	◯	◯	◯	◯	◯	◯
Smoking	◯	◯	◯	◯	◯	◯

126. The person I care for finds it acceptable for me to talk with them about their health behaviours.
Please choose the appropriate response for each item:

	**Strongly agree**	**Agree**	**Unsure**	**Disagree**	**Strongly disagree**	**Not applicable, not a smoker or not drinking alcohol**
Fruit and vegetable consumption	◯	◯	◯	◯	◯	◯
Physical activity	◯	◯	◯	◯	◯	◯
Alcohol	◯	◯	◯	◯	◯	◯
Smoking	◯	◯	◯	◯	◯	◯

127.I feel confident to talk to the person I care for about their health behaviours.

Please choose the appropriate response for each item:

	**Strongly agree**	**Agree**	**Unsure**	**Disagree**	**Strongly disagree**	**Not applicable, not a smoker or not drinking alcohol**
Fruit and vegetable consumption	◯	◯	◯	◯	◯	◯
Physical activity	◯	◯	◯	◯	◯	◯
Alcohol	◯	◯	◯	◯	◯	◯
Smoking	◯	◯	◯	◯	◯	◯

128. I have the knowledge and skills to encourage healthy behaviours for the person I care for.

Please choose the appropriate response for each item:

	**Strongly agree**	**Agree**	**Unsure**	**Disagree**	**Strongly disagree**	**Not applicable, not a smoker or not drinking alcohol**
Fruit and vegetable consumption	◯	◯	◯	◯	◯	◯
Physical activity	◯	◯	◯	◯	◯	◯
Alcohol	◯	◯	◯	◯	◯	◯
Smoking	◯	◯	◯	◯	◯	◯

129. My encouraging healthy behaviours for the person I care for may harm our relationship.
Please choose the appropriate response for each item:

	Strongly agree	Agree	Unsure	Disagree	Strongly disagree	Not applicable, not a smoker or not drinking alcohol
Fruit and vegetable consumption	◯	◯	◯	◯	◯	◯
Physical activity	◯	◯	◯	◯	◯	◯
Alcohol	◯	◯	◯	◯	◯	◯
Smoking	◯	◯	◯	◯	◯	◯

Experience of Smoke Free Policy

This section of the survey contains questions about smoking bans and treatment for smoking provided within inpatient and community mental health settings. Currently total smoking bans exist in all health care facilities including mental health facilities.

130. Total smoking bans in **public places** such as public transport, shopping centres and restaurants are a good thing.

Strongly agree	Agree	Unsure	Disagree	Strongly disagree
◯	◯	◯	◯	◯

131. Total smoking bans in **general hospitals** are a good thing.

Strongly agree	Agree	Unsure	Disagree	Strongly disagree
◯	◯	◯	◯	◯

132. Total smoking bans in **mental health hospitals** are a good thing.

Strongly agree	Agree	Unsure	Disagree	Strongly disagree
◯	◯	◯	◯	◯

133. Total smoking bans in **other mental health treatment facilities** (e.g. community mental health services) are a good thing.

Strongly agree	Agree	Unsure	Disagree	Strongly disagree
○	○	○	○	○

134. Smoking bans in a mental health hospital need to be properly put in place so that no smoking actually occurs.

Strongly agree	Agree	Unsure	Disagree	Strongly disagree
○	○	○	○	○

135. Smoking bans in a mental health hospital need to include treatment for smokers such as Nicotine Replacement Therapy (NRT).

Strongly agree	Agree	Unsure	Disagree	Strongly disagree
○	○	○	○	○

136. Is there anything you would like to comment on regarding your personal experience, or the person you care for, of smoking bans in inpatient and community mental health settings?

____

____

____

____

____

____

____

____

____

137. Is there anything you would like to comment on regarding your personal experience, or the person you care for, of smoking treatment (e.g. Nicotine Replacement Therapy (NRT)) in inpatient and community mental health settings?

____

____

____

____

____

____

____

____

____

____

____

Please answer the following questions about **yourself.**

138. This question asks about how you have been feeling in the ***last four weeks***.

Please tick the appropriate response for **each** item:
In the last four (4) weeks…

	**None of the time**	**A little of the time**	**Some of the time**	**Most of the time**	**All of the time**
About how often did you feel so depressed nothing could cheer you up?	◯	◯	◯	◯	◯
About how often did you feel hopeless?	◯	◯	◯	◯	◯
About how often did you feel restless or fidgety?	◯	◯	◯	◯	◯
About how often did you feel that everything was an effort?	◯	◯	◯	◯	◯
About how often did you feel worthless?	◯	◯	◯	◯	◯
About how often did you feel nervous?	◯	◯	◯	◯	◯

139. What is **your** age?

140. Gender?

- ○ Female
- ○ Male

141. What is **your** current employment status?
Please choose **only one** of the following:

- ○ Employed full time
- ○ Employed part time or casual
- ○ Not currently employed- but seeking employment
- ○ Not currently employed- not seeking employment

142. Are **you** of Aboriginal or Torres Strait Islander origin?
Please choose **only one** of the following:

- ○ Yes, Aboriginal origin
- ○ Yes, Torres Strait Islander origin
- ○ Yes, both Aboriginal and Torres Strait Islander origin
- ○ No

143. What is **your** present marital status?
Please choose **only one** of the following:

- ○ Never married
- ○ Married or living together in a relationship
- ○ Divorced/ Separated
- ○ Widowed

144. What is the highest level of education **you** have achieved?

Please choose **only one** of the following:

- ○ No formal schooling or attended primary school only
- ○ Some high school with less than four years completed
- ○ School certificate, Intermediate, Year 10, 4th Form
- ○ Completed HSC, Leaving, Year 12 or 6th Form
- ○ TAFE certificate or diploma
- ○ University, College of Advanced Education, Degree or higher

145. What is **your** postcode? [ ]

146. Have **you** ever been diagnosed with a mental illness?
Please choose **only one** of the following:

- ○ Yes
- ○ No *(Skip question 147. Go to question 148 on page 44)*

147. What was, or is your primary psychiatric diagnosis?
Please choose **only one** of the following:

- ○ Schizophrenia
- ○ Depression
- ○ Anxiety disorder
- ○ Panic disorder
- ○ Bipolar disorder
- ○ Post-traumatic stress disorder
- ○ Eating disorder
- ○ Personality disorder
- ○ Dementia
- ○ Unsure
- ○ Other: Please describe:

The questions in this section are about **<u>your own physical health behaviours</u>**.

It will contain questions about smoking, fruit and vegetable consumption, alcohol consumption and physical activity.

<u>Please answer these questions about yourself.</u>

148. How many serves of vegetables do you usually eat each day?

One serve of vegetables or legumes is equal to: ½ cup green leafy vegetables like cabbage, spinach, Brussels sprouts or cauliflower; ½ cup green beans, zucchini, mushrooms, turnips, swede or eggplant; 1 cup salad vegetables such as tomato, capsicum and celery; 1 medium sized potato or parsnip.

Please choose **only one** of the following:

- ○ 0
- ○ 1
- ○ 2
- ○ 3
- ○ 4
- ○ 5 or more
- ○ Unsure

149. How many serves of fruit do you usually eat each day?

One serve of fruit is equal to: 150 grams (5.29 oz.) of fresh fruit or; one medium sized fruit (e.g. apple); 2 smaller pieces (e.g. apricots); 1 cup canned or chopped fruit; ½ cup/ 125 ml (5 fl oz.) 100% fruit juice; 1.5 tablespoon dried fruit (e.g. sultanas or 4 dried apricot halves).

Please choose **only one** of the following:

- ○ 0
- ○ 1
- ○ 2 or more
- ○ Unsure

150. How many days a week do you usually do 30 minutes or more of physical activity?

By physical activity we mean any activity that increases you heart rate or makes you breathe harder than normal. This can include brisk walking, swimming, team sports or even things like gardening. You can add up your total time during the day, for example walking to the shops and back.

Please choose **only one** of the following:

- ◯ 0
- ◯ 1
- ◯ 2
- ◯ 3
- ◯ 4
- ◯ 5
- ◯ 6
- ◯ 7, everyday
- ◯ Unsure
- ◯ Can't do physical activity for health or treatment reasons

151. Do you live in a smoke-free household?

By this we mean people may be smokers but no smoking is permitted inside the residence.

Please choose **only one** of the following:

- ◯ Yes
- ◯ No
- ◯ Unsure

152. Are you a smoker of any tobacco products?

This could include 'roll your own', cigars, pipe etc.

Please choose **only one** of the following:

- ◯ Yes, daily *(go to the next question)*
- ◯ Yes, at least once a week *(go to the next question)*
- ◯ Yes, less than once a week *(go to the next question)*
- ◯ No, trying to quit *(go to question 160, on page 47)*
- ◯ No, quit longer than 4 months ago *(go to question 160, on page 47)*
- ◯ No, never smoked *(go to question 161, on page 48)*

153. How many cigarettes are you usually smoking each day?
Please choose **only one** of the following:

- ○ 10 or less
- ○ 11 to 20
- ○ 21 to 30
- ○ 31 or more
- ○ Unsure

154. How soon after waking are you usually having your first smoke?
Please choose **only one** of the following:

- ○ Within 5 minutes
- ○ 6 to 30 minutes
- ○ 31 to 60 minutes
- ○ More than 60 minutes
- ○ Unsure

155. In the last year, did you ever on purpose quit smoking for at least 24 hours?
Please choose **only one** of the following:

- ○ Yes
- ○ No
- ○ Unsure

156. Have you ever made an attempt to quit smoking in the past?
Please choose **only one** of the following:

- ○ Never *(skip question 157, go to question 158 on page 47)*
- ○ Once
- ○ 2 to 4 attempts
- ○ 5 or more attempts
- ○ Unsure *(skip question 157, go to question 158 on page 47)*

157. How long ago was your last quit attempt?
Please choose **only one** of the following:

- ○ Currently trying to quit
- ○ 3 months or less
- ○ Between 3 and 12 months ago
- ○ More than one year ago
- ○ Unsure

158. Do you plan to quit smoking?
Please choose **only one** of the following:

- ○ Yes
- ○ No *(Skip question 159, go to question 160, on this page)*
- ○ Unsure *(Skip question 159, go to question 160, on this page)*

159. When do you plan to quit smoking?
Please choose **only one** of the following:

- ○ Within the next month
- ○ Within the next 2 to 6 months
- ○ More than 6 months
- ○ Unsure

160. Which of the following have you ever used in an attempt to quit smoking?
Please choose **all** that apply:

- ○ Nicotine Replacement Therapy (NRT); such as patches or gum
- ○ E cigarette
- ○ Quitline
- ○ GP (doctor) advice
- ○ Hypnosis
- ○ Acupuncture
- ○ Champix (Varenicline)
- ○ Zyban (Buproprion)
- ○ Other medications
- ○ 'Cold turkey' (I just stopped on my own with no assistance)
- ○ None of these

161. How often do you have a drink containing alcohol?
Please choose **only one** of the following:

- ○ Never, not drinking alcohol *(go to question 164 on page 49)*
- ○ Monthly or less
- ○ 2 to 4 times a month
- ○ 2 to 3 times a week
- ○ 4 or more times a week
- ○ Unsure

162. How many standard drinks would you have on a typical drinking day?

Please refer back to the diagrams on pages 11 and 12 as a guide of standard drink measurements.
Please choose **only one** of the following:

- ○ 1 to 2
- ○ 3 to 4
- ○ 5 to 6
- ○ 7 to 9
- ○ 10 or more
- ○ Unsure

163. How often would you have four or more standard drinks on one occasion?
Please choose **only one** of the following:

- ○ Never
- ○ Less than monthly
- ○ Monthly
- ○ Weekly
- ○ Daily or almost daily
- ○ Unsure

164. In the last year, have you had an interest in improving any of your own health behaviours?

Please choose the appropriate response for **each** item:

You may want to answer Not Applicable for "Alcohol" or "Smoking" if the person you care for is not a smoker or does not drink alcohol.

	**Yes**	**No**	**Unsure**	**Not Applicable, not a smoker or not drinking alcohol**
Fruit and vegetable consumption	◯	◯	◯	◯
Physical activity	◯	◯	◯	◯
Alcohol	◯	◯	◯	◯
Smoking	◯	◯	◯	◯

165. Do you think any of the following factors are health risks for you?

Please choose the appropriate response for **each** item:

	**Yes**	**No**	**Unsure**	**Not Applicable, not a smoker or not drinking alcohol**
Not eating enough fruit and vegetables	◯	◯	◯	◯
Not doing enough physical activity	◯	◯	◯	◯
Alcohol use	◯	◯	◯	◯
Smoking	◯	◯	◯	◯

166. Can you please tell us roughly how long it took you to complete this survey?

In minutes.

167. Any comments you would like to make on the survey would be appreciated. Was it easy? Hard? Were any questions difficult to understand? If you have any ideas on how to improve the survey that would be greatly appreciated. Thank you.

____

____

____

____

____

____

____

____

168. We may want to ask you some questions based on your responses to this survey and similar issues discussed within this survey.

If you are comfortable with this, you can check the box below that says "I consent to being contacted through my organisation" where we will ask your organisation (the organisation that you received this survey from) to contact you. **If you check the box we will *not* gain access to your personal information,** your organisation will contact you and provide you with the details of what we want to discuss with you and you can respond if you wish.

☐ I consent to being contacted through my organisation in the future.

Thank you very much for taking the time to complete this survey.

If you have any questions regarding the survey you can contact the lead investigator Jenny Bowman by email at Jenny.Bowman@newcastle.edu.au or by telephone: 49215958. Alternatively, you can ask any questions through your support organisation.

If answering any of the questions in the survey has left you feeling upset, worried or concerned you can contact Lifeline on 131114 or Beyondblue on 1300224636. Lifeline and Beyondblue are telephone helplines where you can talk to someone about how you are feeling. You can call these numbers and talk to someone 24 hours a day, 7 days a week.
